# Composition-Based Risk Estimation of Mycotoxins in Dry Dog Foods

**DOI:** 10.3390/foods12010110

**Published:** 2022-12-25

**Authors:** Ovidiu Ionut Geicu, Liviu Bilteanu, Loredana Stanca, Adriana Ionescu Petcu, Florin Iordache, Aurelia Magdalena Pisoschi, Andreea Iren Serban

**Affiliations:** 1 Department of Preclinical Sciences, Faculty of Veterinary Medicine, University of Agronomic Sciences and Veterinary Medicine of Bucharest, 105 Blvd. Splaiul Independentei, 050097 Bucharest, Romania; 2 Molecular Nanotechnology Laboratory, National Institute for Research and Development in Microtechnologies, 126A, Erou Iancu Nicolae Street, 077190 Bucharest, Romania; 3 Department of Biochemistry and Molecular Biology, Faculty of Biology, University of Bucharest, 91–95 Blvd. Splaiul Independentei, 050095 Bucharest, Romania

**Keywords:** risk estimation, dry dog food, cereal, mycotoxin

## Abstract

The risk of mycotoxins co-occurrence in extrusion-produced dry foods increases due to their composition based on various grains and vegetables. This study aimed to validate a risk estimation for the association between ingredients and the ELISA-detected levels of DON, FUM, ZEA, AFs, T2, and OTA in 34 dry dog food products. The main ingredients were corn, beet, and oil of different origins (of equal frequency, 79.41%), rice (67.6%), and wheat (50%). DON and FUM had the strongest positive correlation (0.635, *p* = 0.001). The presence of corn in the sample composition increased the median DON and ZEA levels, respectively, by 99.45 μg/kg and 65.64 μg/kg, *p* = 0.011. In addition to DON and ZEA levels, integral corn presence increased the FUM median levels by 886.61 μg/kg, *p* = 0.005. For corn gluten flour-containing samples, DON, FUM, and ZEA median differences still existed, and OTA levels also differed by 1.99 μg/kg, *p* < 0.001. Corn gluten flour presence was strongly associated with DON levels > 403.06 μg/kg (OR = 38.4, RR = 9.90, *p* = 0.002), FUM levels > 1097.56 μg/kg (OR = 5.56, RR = 1.45, *p* = 0.048), ZEA levels > 136.88 μg/kg (OR = 23.00, RR = 3.09, *p* = 0.002), and OTA levels > 3.93 μg/kg (OR = 24.00, RR = 3.09, *p* = 0.002). Our results suggest that some ingredients or combinations should be avoided due to their risk of increasing mycotoxin levels.

## 1. Introduction

The global food crisis, food safety regulations, the change in the human lifestyle that prefers ready-to-eat foods, and the pet industry’s development have led to the large-scale use of extrusion technology in food industries in recent years. Extrusion of food technology has led to the production of a wide variety of products such as pasta, breakfast cereals, bread crumbs, biscuits, crackers, croutons, baby foods, snack foods, confectionery items, texturized vegetable protein, modified starch, dry pet foods, dried soups, dry beverage mixes, etc., [1]. Extrusion is an ideal processing method for manufacturing affordable long shelf-life foods containing cereals (corn, wheat, rice, wheat, oats, and others), fibers, and vegetable oils. The beneficial nutritional effects of extruded foods range from increased protein and starch digestibility to the retention of various micronutrients [2]. On the other hand, food safety concerns have become one of our most important problems, and the fungal infection and their resulting secondary metabolites (mycotoxins) that affect crops are significant problems aggravated by climate change [1,2]. However, extrusion is a technology that seems to reduce the content of mycotoxins in dry products or cereals [3,4], and few studies support this fact [5,6].

Moreover, mycotoxins are generally resistant to thermal treatments. Depending on the applied process, their presence is reduced but not eliminated [7]. Cereals and vegetables are highly susceptible to such types of infection during their production’s pre-harvest and post-harvest stages [4]. The fungi belonging to the genera Fusarium, Alternaria, and Cladosporium contaminate cereals in the field (moisture content of 18–30%), while Aspergillus, Penicillium, and Mucor are contaminants of cereals in storage conditions (moisture content of 14–16%) [8,9,10]. These fungal genera include many mycotoxin-producing species. When ingested, inhaled, or cutaneously absorbed, mycotoxins can cause acute and chronic disorders due to their immune toxicity, carcinogenicity, hepatotoxicity, nephrotoxicity, mutagenicity, and teratogenicity [4,8]. The most studied mycotoxins contaminating foods and feeds are aflatoxins (AFs) such as AFB1, AFB2, AFG1, AFG2, and AFM1, ochratoxin (OTA), trichothecenes including deoxynivalenol (DON), nivalenol (NIV), T-2 toxin (T2) and HT-2 toxin (HT-2), zearalenone (ZEA), fumonisins (FBs: FB1, FB2, FB3, and FB4), moniliformin (MON), and beauvericin (BEA) [11,12,13,14]. Their occurrence and presence in a specific food or feed product depend on both extrinsic factors associated with environmental conditions fluctuation (temperature and relative humidity), microbial, insect damages, and mechanical injuries and intrinsic factors relating to the applied fabrication technology (moisture content, pH) and the composition of the foods [4,8,15,16,17]. In general, dry food products have a variable composition in grains, so the risk of the co-occurrence of several types of mycotoxins in a particular product is increased. Moreover, cereal crops in the field or during storage can be contaminated with more than one species of fungi, and thus the co-occurrence of mycotoxins in dry food products is difficult to manage. The identification and quantification of one single mycotoxin in a dry food product is an exception, and both humans and animals are usually exposed to several mycotoxins, mainly at low levels, at the same time [11,18,19]. The frequent mycotoxin combinations in grain-containing foods described in the literature are AFs + FUM, DON + ZEA, AFs + OTA, and FUM + ZEA [11,20,21,22,23,24]. However, only a few studies specified the number of co-occurring mycotoxins with the percentage of the co-contaminated samples, as well as the main combinations found in relation to food composition. In addition, the regulations regarding the maximum allowed limit of mycotoxins in food, feed, and cereals take into account individual mycotoxins and not their combinations [25,26,27,28,29,30,31,32]. A meta-analysis concerning the toxicological effect of mycotoxin combinations in vitro and in vivo classified their interactions into synergistic, additive, or antagonistic effects. This analysis also highlighted the complexity of mycotoxins interactions which varies according to the dose of toxins combinations, the animal species, and the duration of exposure [33]. 

Chromatographic techniques are by far the most common analytical methods for the quantification of mycotoxins in food and feed, mainly thin layer chromatography (TLC) and high-performance liquid chromatography (HPLC), gas chromatography (GC) in combination with various detectors such as diode array, fluorescence, and UV [34,35,36]. However, the liquid chromatography-tandem mass spectrometry (LC-MS/MS) technique remains essential for detecting numerous mycotoxins, ensuring high precision, sensitivity, reproducibility, and a low detection limit and allowing simultaneous detection of different mycotoxins, regardless of their chemical structure [37,38,39]. LC-MS/MS and HPLC have been validated for mycotoxins detection in cereals and their by-products, but they are time-consuming methods requiring expensive and sophisticated equipment and specialized staff. [34,35]. If a rapid and sensitive on-site analysis would be required, for example, at a food production site, in the storehouse, or the granary, immunoassay-based methods such as ELISA and lateral flow immunoassay are suitable options [40,41,42,43]. Thus, ELISA can be used for primary screening because it is a robust and easy-to-handle method that allows quantitative determination of mycotoxins in food and feed with low costs and simple sample preparation with limited organic solvents. However, cross-reactivity with related mycotoxins and matrix interference could lead to the under- or overestimation of mycotoxin contents in samples [35,42].

Standard statistical tests such as one-sample *t*-tests, median comparisons, chi-squared tests for crosstabs, and binary logistic regressions are prevalent in food science literature [44]. They are used to determine potential factors leading to changes in mycotoxin levels. However, models such as linear regression, analysis of variance (ANOVA), analysis of covariance (ANCOVA), and general linear models (GLM), which are very similar in all practical applications, are restrained [44], while risk models have never been employed.

In this study, we analyzed samples of dry dog food from three different manufacturers sold in dedicated stores in Bucharest, Romania. We focused on the description and validation of a statistical analysis methodology by estimating the risk parameters of the association between some ingredients and the levels of mycotoxins that are more frequently reported in the literature, namely DON, FUM, ZEA, AFs, T2, and OTA. The calculated risk parameters, odds ratios (OR), and risk ratios (RRs) associate the increase in mycotoxins above certain levels with the presence of a particular ingredient.

## 2. Materials and Methods

### 2.1. Samples

The material consisted of dry dog foods formulated as croquettes of different sizes, produced by three different manufacturers (we have labeled as A, B, and C), and available in the Romanian market. The samples were purchased in the original hermetically sealed packages of 1.5–3 kg. The research material collected was represented by 34 samples. The expiry date was considered, and no sample expiring before 4 months was purchased. Samples were grouped according to the producer and the type of targeted disease: metabolic conditions (MC), allergies conditions (AC), and gastrointestinal conditions (GC). Each manufacturer has a range with several products for each dietary category (Table 1). The main ingredients (grains and plant products) for each diet range (all producers combined) are presented in Table 2. 

### 2.2. Samples Preparation

The samples of dry dog food material of various sizes were homogenized before analysis in a ceramic mortar and then milled. Packages containing dry dog food were kept sealed and opened only when the quantification procedures started. All of the contents of the original dry dog food package (1.5–3 kg) were split and divided into 16 equal parts (12 cm × 12 cm) on a perfectly clean surface (50 cm × 50 cm) covered with aluminum foil. Then, a few dry dog food pieces are taken from each surface in a beaker. From each beaker containing dry dog food pieces, 20 g were weighed and crushed to a fine powder in a porcelain mortar until 1 mm particles were obtained. Each type of sample was processed in triplicate.

### 2.3. Mycotoxins Detection and Quantification

The quantification of the mycotoxins AFs, DON, FUM, OTA, T2 toxin, and ZEA in dry dog foods was performed with kits produced by Romer Labs (Austria): AgraQuant Total AFtoxin 1/20 ELISA kit having a limit of detection (LOD) of 1 µg/kg and a limit of quantification (LOQ) of 1 µg/kg, AgraQuant Fumonisin 0.25/5.0 ELISA kit with an LOD of 0.2 µg/kg and LOQ of 0.25 µg/kg, AgraQuant Deoxynivalenol 250/5000 ELISA kit with an LOD of 200 µg/kg and LOQ of 250 µg/kg, AgraQuant Ochratoxin 2/40 ELISA kit with an LOD of 1.9 µg/kg and LOQ of 2 µg/kg, AgraQuant T-2 Toxin 20/500 ELISA kit with an LOD of 10 µg/kg and LOQ of 20 µg/kg, and AgraQuant Zearalenone Plus 25/1000 ELISA kit with an LOD of 20 µg/kg and LOQ of 25 µg/kg.

The extraction and quantification of the mycotoxins in the cereals were carried out according to the manufacturer’s instructions. Briefly, AFs, FUM, OTA, T2 toxin, and ZEA were extracted in 70% methanol in water (extraction ratio 1:5), and DON was extracted in distilled water (extraction ratio 1:10). In a blender, the samples were homogenized three times for 1 min at a speed of 10,000 rpm. After 2 h of 4 °C extractions, the samples were centrifuged at 4 °C for 30 min at 5000 rpm. The tubes were kept at 4 °C for one hour to solidify the lipid layer, and the supernatants were carefully collected. A volume of 100 µL of each extract was used for the direct competitive ELISA procedure. Spectrometric analysis was performed using a microplate reader ((PR 4100 Absorbance Microplate Reader, software Magellan, Bio-Rad, Hercules, CA, USA) at 450 nm and with a differential filter at 630 nm. The recovery range was evaluated using a quality control material—corn naturally contaminated with mycotoxins (AFs, OTA, ZEA, FUM, DON, and T2/HT2 toxin) (Trilogy Analytical Laboratory, Washington, DC, USA). For DON, the recovery ranged from 86.8 to 110% with a coefficient of variation (CV) of 0.4 to 2.4%. In the case of FUM, the recovery ranged from 99.0 to 120%, and the CV ranged from 1.0 to 4.9%, and for ZEA, they were 95.8 to 108.6% and 1.2 to 4.5%. For OTA, the recovery varied from 90.0 to 91.7% with a CV of 4.8 to 8.9%; for T2, the recovery ranged from 97.2 to 110.6% with a CV of 1.0 to 1.9%. AFs had a recovery interval of 86.8–120% with a CV of 0.1–8.9%.

### 2.4. Statistical Processing

#### 2.4.1. Variables

The first step of the analysis was to define the basic ingredient-related statistical variables by classifying the ingredients into classes called primary list, secondary list, and combinations list according to product labels (Table 3). Within the primary list, we have included the following source ingredients enumerated as such or retrieved indirectly from the product labels and listed in the heading of Table 2. The secondary list included derived ingredients from the sources in the primary list or some subtypes of these primary ingredients. From the primary list, only corn, wheat, rice, and oil exhibited corresponding ingredients in the secondary list, as seen in Table 1.

**Figure 1:**
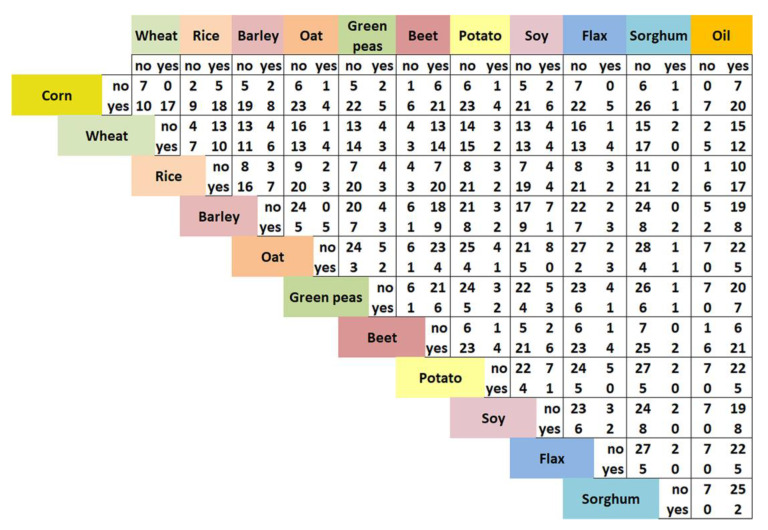
Two by two crosstabs of main ingredients in the composition of the analyzed samples (all diets and producers combined). The pairwise manner account for the presence of the ingredients’ source in the simultaneous presence (yes-yes), the presence of one source while the other is absent (yes-no or no-yes), and the concomitant absence (no-no).

Ingredients susceptible to contamination were found in combinations of two or more in the composition of the analyzed products. Thus, in the second stage of our analysis, we described the concomitance of the main ingredients in the analyzed products. We defined a new set of ingredient-related variables describing the pairwise concomitance of ingredients in the primary list (see Figure 1) and all combinations of primary list ingredients (except corn, wheat, rice, and oil) and the elements of the secondary list composed of derived ingredients or subclasses of ingredients (related to corn, wheat, rice, and oil). For example, one particular combination of integral corn and corn gluten flour was present on the labels of several products. The primary and secondary ingredient-related qualitative variables are listed and described in Table 4, with their respective notations and examples.

In addition to the ingredient-related variables, we defined mycotoxin level-related variables. The primary variables, $t_{\ell }$ (ℓ is indexing the mycotoxins listed in the column heads of Table 5) were the mycotoxin levels as they were measured using the methodology in Section 2.3. The descriptive statistics of these primary variables are presented in Table 5.

The quantitative variables, $t_{\ell }$ can be converted into quantitative variables using their percentiles. For example, for ℓ=1 (i.e., ℓ=‘DON’), the 25th percentile is $t_{1}^{25\%}=331.88$. For all samples with $t_{\ell }\leq t_{1}^{25\%}$, the secondary variable $t_{1}^{*25\%}=0$, otherwise $t_{1}^{*25\%}=1$. The choice for α values may not be limited to 25%, 33%, 50%, 67%, or 75% and can be expanded to a more extensive set of percentiles. 

#### 2.4.2. Mycotoxin Co-Occurrence

Each variable was tested to see if it follows a Gaussian (normal) distribution by applying the Kolmogorov–Smirnov (KS) test. The KS test showed that all $t_{\ell }$ variables were not normally distributed. Hence, the correlations between the levels of different types of mycotoxins were represented through Spearman correlation coefficients, whose statistical significance threshold was considered *p* < 0.05. Correlation coefficients are a measure of mycotoxins co-occurrence in samples.

#### 2.4.3. Ingredients’ Impact on Mycotoxin Levels—Comparisons and Risk Analysis

The identification of ingredients (individual or in combinations) generating statistically significant differences between mycotoxin levels was achieved by comparing median mycotoxins levels ($m_{\ell }$) between sample groups defined through the belonging (for example $x_{i}^{\left(1\right)}=1$) or not (i.e., in the case of the example $x_{i}^{\left(1\right)}=0)$ of each ingredient in the composition reported on the label. The null hypothesis (H0) is $m\left[x_{i}^{\left(1\right)}=1\right]=m\left[x_{i}^{\left(1\right)}=0\right]$ (equality of medians). The median test allowed us to reject or accept H0. If H0 is rejected (*p* < 0.05), we may conclude that the ingredient indexed by i determines statistically significant differences between the groups thus defined. The procedure was followed independently if the ingredient belonged to the primary (indexed by i) or secondary (indexed by j) list or if we dealt with a combination of ingredients on these lists (indexed by *k*). Once the difference was established, we checked the difference $\Delta m\left(x_{i}^{\left(1\right)}\right)=m\left[x_{i}^{\left(1\right)}=1\right]-m\left[x_{i}^{\left(1\right)}=0\right]$. Only the ingredients and combinations for which $\Delta m\left(x_{i,j,k}^{\left(1,2,3\right)}\right)>0$ were retained for further investigations

Finally, the identified associations between ingredients and mycotoxins were refined through risk estimations by odds ratios (OR), Equation (1) and relative risks (RR), Equation (2):

(1) $$\mathrm{OR}\;\left(\left\{i,j,k\right\},\ell ,\alpha \right)=\frac{N\left(t_{\ell }>t_{\ell }^{\alpha }|x_{i,j,k}^{\left(1,2,3\right)}=1\right)/N\left(t_{\ell }\leq t_{\ell }^{\alpha }|x_{i,j,k}^{\left(1,2,3\right)}=1\right)}{N\left(t_{\ell }>t_{\ell }^{\alpha }|x_{i,j,k}^{\left(1,2,3\right)}=0\right)/N\left(t_{\ell }\leq t_{\ell }^{\alpha }|x_{i,j,k}^{\left(1,2,3\right)}=0\right)}$$

(2) $$RR\left(\left\{i,j,k\right\},\ell ,\alpha \right)=\frac{N\left(t_{\ell }>t_{\ell }^{\alpha }|x_{i,j,k}^{\left(1,2,3\right)}=1\right)/N\left(x_{i,j,k}^{\left(1,2,3\right)}=1\right)\;}{N\left(t_{\ell }>t_{\ell }^{\alpha }|x_{i,j,k}^{\left(1,2,3\right)}=1\right)/N\left(x_{i,j,k}^{\left(1,2,3\right)}=0\right)}$$

In Equations (1) and (2), we denoted by N, the number of samples that met the condition in parentheses. So $x_{i,j,k}^{\left(1,2,3\right)}=1$ signifies the presence of the ingredient indexed with one of the indices i, j or k, and $x_{i,j,k}^{\left(1,2,3\right)}=0$ signifies the absence of such an ingredient. Also $t_{\ell }>t_{\ell }^{\alpha }$ indicates that the level of mycotoxin ℓ is greater than $t_{\ell }^{\alpha }$ and $t_{\ell }\leq t_{\ell }^{\alpha }$ indicates the opposite. The juxtaposition of two conditions separated by a vertical bar indicates the simultaneity of their compliance. Thus, for example, $N\left(t_{\ell }>t_{\ell }^{\alpha }|x_{i,j,k}^{\left(1,2,3\right)}=1\right)$ stands for the number of samples for which the level of mycotoxin ℓ is greater than the $t_{\ell }^{\alpha }$ value, provided that these samples are known to contain the index active ingredient with one of the indices i, j, or k ($x_{i,j,k}^{\left(1,2,3\right)}=1$).

These parameters highlighted the magnitude of associations between these ingredients or their combinations (the qualitative variables $x_{i,j,k}^{\left(1,2,3\right)}$) and mycotoxin levels (represented by the secondary variables, $t_{\ell }^{\alpha }$, defined for various percentiles). The statistical significance of these associations was computed using a logistical univariate model linking $t_{\ell }^{\alpha }$ and $x_{i,j,k}^{\left(1,2,3\right)}$, and p<0.05 were considered statistically significant. For each $x_{i,j,k}^{\left(1,2,3\right)}$, OR and RR are dependent on $t_{\ell }^{\alpha }$ (the values of percentiles). Plots of this dependence for each mycotoxin (i.e., for each index ℓ) constitute predictive models linking the composition to mycotoxin levels. A coherence criterion linking an ingredient or a combination to a mycotoxin would be a monotonous relationship (increasing or decreasing) between $t_{\ell }$ and OR values. The ideal plot for fulfilling such a criterion would exhibit as many pairs ($t_{\ell }^{\alpha },\;OR$).

## 3. Results

Mycotoxin levels were analyzed in the first stage, starting from the influence of the main ingredients in the composition reported on the label individually and in combinations, as presented in Table 3. The most common main ingredients were corn, beets, and oil of different origins (each one found in 79.41% of the samples), rice (67.6%), and wheat (50%). These ingredients were also found in combinations in the following frequencies: beet with oils of different origins or beet with corn in different forms were the most frequent combinations (61.8% of the studied samples). The other two most frequent combinations (58.8%) were rice with beet and corn with oil. Rice is also found in subproducts with corn (in 52.9% of the samples) Figure 1. The combinations of corn with wheat and rice with oil were present in half of the samples. As can be seen from Figure 1, all of the samples that contain wheat also contain corn in different forms (17), all the samples that contain oats also contain barley (5), and all that contain corn in different forms also contain an oil from different origins (20).

The levels of the mycotoxins identified in the studied samples are presented synthetically by the descriptive statistics in Table 5. The recommended mycotoxin limits are also presented in this table [32]. The average values of these mycotoxins did not exceed the recommended limits. However, the recommended levels for ZEA, AFs, and FUM were exceeded in 41.2%, 35.3%, and 2.9% of the samples.

The paired correlations of the studied mycotoxin levels are positive, with few exceptions being significant (Table 6). The most substantial and statistically significant correlation was between DON and FUM (0.635, *p* < 0.001). It should be noted that DON was correlated with all other mycotoxins studied (DON and ZEA, 0.510 and *p* = 0.002; DON and T2, 0.481, *p* = 0.004; DON and OTA, 0.343 and *p* = 0.047), and FUM was also correlated with all mycotoxins except T2. ZEA also had numerous correlations with all mycotoxins except OTA, with the highest and most statistically significant correlation coefficients corresponding to DON and FUM (0.507, *p* = 0.002). In addition, OTA correlated to the same extent with FUM and T2 (0.436, *p* = 0.01) and less with DON (0.343, *p* = 0.047).

**Figure 2:**
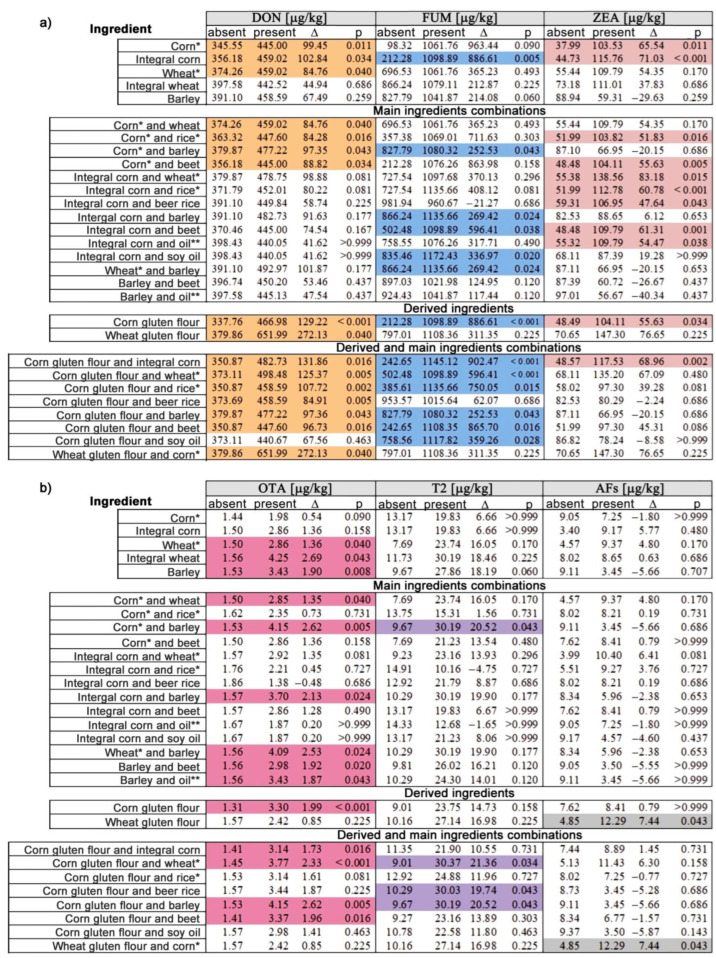
Positive and statistically significant differences in the median levels of (**a**) DON, FUM, and ZEA and (**b**) OTA, T2, and AFs mycotoxins in groups defined (absence or presence) of some main ingredients, some derived ingredients, or their combinations in the composition of the samples. The statistically significant differences for DON are highlighted in orange, for FUM in blue, for ZEA in pale pink, for OTA in pink, for T2 toxin in purple, and AFs in gray. * All derived ingredients included; ** All origins included.

The mycotoxins levels were compared across groups defined by the presence of the main ingredients, some derivatives of these ingredients, or their combinations (Figure 2). DON and ZEA levels were higher in samples containing corn (Figure 2a). Moreover, the differences were more significant if integral corn was present in the composition. The presence of integral corn was also associated with higher levels of FUM (Figure 2a). DON and OTA levels were higher in samples containing wheat. These differences were more significant with OTA when integral wheat was listed as an ingredient (Figure 2b). The presence of barley was also associated with higher levels of OTA (Figure 2b). Corn gluten flour was the derived ingredient that we found to have a decisive role in increasing the levels of DON, FUM, ZEA, and OTA, while wheat gluten flour increased DON and AFs levels (Figure 2a,b).

Among the ingredients derived from corn, corn gluten flour determined the highest and most statistically significant differences in the case of DON and FUM (Figure 2a). This was also the only derived ingredient associated with significant differences (*p* < 0.001) in OTA levels (Figure 2b). The highest differences in DON levels were identified in wheat gluten flour-containing samples. This derivative ingredient was the only one among all the main ingredients, derivatives, or combinations thereof that was decisively associated with significantly higher levels of AFs (Figure 2b). Samples containing corn in all its forms combined with wheat (in all its forms), rice, oats, and beet led to a slight decrease in DON levels. The decreases were more significant in the case of ZEA levels when corn was present along with rice and beet (Figure 2a). On the other hand, OTA levels increased (through an additive effect) when corn was combined with barley. This association led to the emergence of significant differences in T2 levels (Figure 2b). 

The statistically significant difference in the DON levels determined by the presence of integral corn was no longer found when this ingredient was in combination with wheat in various forms, rice (in particular beer rice), barley, beet, or oil (in particular soybean oil). The same situation was highlighted in the case of FUM when integral corn was combined with wheat in different forms, rice (in particular beer rice), or oils of different origins (Figure 2a). Combinations with barley, beet, or soybean oil significantly decreased FUM difference values. Except for the products containing integral corn combined with wheat in various forms that cause increases in ZEA levels, products containing combinations of integral corn with the other ingredients mentioned above were associated with decreases in ZEA levels (Figure 2a). Additive effects on the increase in OTA levels were observable in the case of dog food products containing combinations of wheat or corn with barley in different forms. Combinations of barley with beet or oils of different origins did not significantly change the levels of this mycotoxin, highlighting that OTA may potentially have originated from barley (Figure 2b). DON difference values were most reduced when corn gluten flour was combined with beer rice or beet (Figure 2a). When corn gluten flour was combined with soy oil, the FUM difference levels were significantly reduced (about 2.5 times compared to the difference in the absence of soy oil) (Figure 2a). The co-occurrence of corn gluten flour and integral corn had an additive effect (increase by approximately 20%) on ZEA levels (Figure 2a). Additive effects were also observed in the case of OTA levels in products having combinations of corn gluten flour and barley, of the same magnitude as those determined in the case of the combination of corn of different origins and barley, indicating that OTA in the case of corn originated from corn gluten flour (Figure 2b). The same additive effect was recorded in the case of toxin T2 for the same combinations of ingredients, emphasizing that in these samples, T-2 most likely originated from corn gluten flour and barley. Additive effects in the case of T2-toxin levels were also detected in the case of combinations of corn gluten flour with wheat in various forms or beer rice. (Figure 2b).

To establish an association between the presence of an ingredient from the three categories previously described in Section 2.4.1. and mycotoxin levels above the threshold values that represent percentile values, $t_{\ell }^{\alpha }$, we evaluated OR and RR. 

The presence of corn and wheat correlated punctually with DON levels above 364 µg/kg and 403 µg/kg, respectively (Figure S1a). Furthermore, we found that the correlation with higher levels compared to these threshold values was also the case for integral corn, with the degree of association increasing monotonically with the threshold value reaching the value OR = 10.9 (RR = 6.2, *p* = 0.034) for values of DON levels above 462 µg/kg (Figure S1a).

Combinations of corn with other ingredients in the first category (rice, beet, and wheat) do not significantly alter the levels of associations found in the case of individual corn; most of these associations were achieved with DON levels above 403 µg/kg (Figure S1b). However, it should be noted that for the combination of integral corn and wheat, we found the highest association with DON levels above the 75th percentile, 502 µg/kg (OR = 9.0, RR = 5.0, *p* = 0.017) (Figure S1b).

The combination of corn gluten flour with integral corn has systematically higher degrees of association with DON levels, for which we found statistically significant associations with the presence of integral corn, for example at 459 µg/kg DON (~67th percentile), the OR was 10.9 (RR = 6.173) for integral corn and in the case of the combination OR was 19 (RR = 3.425). Thus, it was observed that the presence of corn gluten flour increased the association with DON at 459 µg/kg but reduced the risk by half (Figure S1c). The combinations of corn gluten flour with rice or beet show significant OR and RR with DON levels around the 50th percentile, and the combination with wheat shows significant OR and RR at all DON threshold values over the 75th percentile (502 µg/kg). The highest value for the association with DON levels (OR = 23.0, RR = 6.5, *p* = 0.002) was reached in the case of the combination of corn from different origins and wheat gluten flour, in the range of the 75th percentile (Figure S1c).

Among all ingredients in category 1 or 2, the presence of corn has a high degree of association (OR = 48, RR = 6.2, *p* = 0.002) with FUM levels above 215 µg/kg, the degree of this association diminishing at the threshold value of the FUM level of 609 µg/kg, corresponding to the 33rd percentile (Figure S2a). Also, worth mentioning were the associations of wheat in various forms, barley, and corn gluten flour with FUM levels above threshold values of 215 µg/kg, 962 µg/kg, and 1098 µg/kg, respectively (Figure S2a). As in the case of DON, the presence of integral corn is systematically associated with FUM levels above several threshold values, with the highest OR and RR reaching above the 50th percentile (962 µg/kg) (Figure S2a). Products containing combinations of corn with wheat or rice decreased the OR and RR levels compared to those determined in the case of products containing only corn at FUM concentrations of 215 and 609 µg/kg, respectively (Figure S2b). The association between corn and beet was a systematic one found for various threshold values of FUM levels, with the highest RR value of 6.173 (*p* = 0.034) above the 67th percentile (Figure S2b). The simultaneous presence of integral corn and corn gluten flour shows monotonically increasing degrees of association with FUM threshold levels of 215, 609, and 962 µg/kg (Figure S2c). This combination’s highest degree of association was achieved with FUM values above the 50th percentile at 962 µg/kg (OR = 35, RR = 6.7, *p* < 0.001) (Figure S2c). The combination of integral corn and beets for the same FUM threshold levels systematically decreased the degree of association (Figure S2c). The combination of corn gluten flour with ingredients other than integral corn (i.e., wheat, rice, barley, and beet) diminished the degree of association with FUM levels above the threshold values of 609 and 962 µg/kg (Figure S2d). The combinations of corn gluten flour and beet or rice remained associated with FUM levels higher than the 75th percentile (1190 µg/kg), with OR and RR statistically more significant than those corresponding to corn gluten flour alone (Figure S2d).

As expected, corn was associated with ZEA levels detected in our samples (Figure 2a). Thus, ZEA levels above a threshold value of 55 µg/kg (the 33rd percentile) were particularly associated with the presence of corn (OR = 32, RR = 5.7, *p* = 0.006) and especially with integral corn (OR = 66.7, RR = 4.1, *p* = 0.001) (Figure S3a). Combinations of corn with rice or beet maintained some associations, but to a lesser degree (the OR decreased) with ZEA levels above the threshold value of 55 µg/kg (Figure S3b). The combination of integral corn with beet was associated to a lower degree (compared to integral corn considered individually) with levels of ZEA above the threshold value of 55 µg/kg (Figure S3c). Although the combinations of integral corn with wheat or beet were associated with ZEA levels above higher threshold values (112 µg/kg, OR = 5.9 and 135 µg/kg, OR = 25), the intensity of these associations did not exceed the association between ZEA and integral corn at threshold values above 55 µg/kg (OR = 66.7). (Figure S3c). The presence of corn from different origins was associated with ZEA levels above the 33rd percentile (55 μg/kg). In combination with wheat gluten flour, the intensity of the association remained approximately the same, but the ZEA levels with which this combination was associated were above the 75th percentile (137 μg/kg) (Figure S3d).

The association of integral corn with OTA levels above 1.44 µg/kg has the same intensity as that of the same ingredient with ZEA levels above the same percentile (33rd) (OR = 66.7, RR = 4.1, *p* = 0.001) (Figure S4a). Wheat and the combination between corn and wheat from different origins had the same magnitude of association (OR = 12.2, RR = 8.0, *p* = 0.02) with OTA levels above 3.93 µg/kg (75th percentile), suggesting that OTA originated mainly from contaminated wheat (Figure S4a). The combination of corn and barley, beet, or rice was associated with OTA levels above the median 1.76 μg/kg, while associations with levels above the 67th percentile (3.12 μg/kg) existed only for the corn with barley ingredient combination. This showed a possible link between barley and higher OTA levels (Figure S4b). The combinations of integral corn and wheat, on one side, and barley and beet on the other were the only combinations that exhibited the highest association with OTA levels about the 75th percentile (3.93 µg/kg) (Figure S4c). Gluten flours independent of origin (corn or wheat) were associated (OR = 23) with OTA levels above the 75th percentile (3.93 μg/kg) (Figure S4d). These results emphasized that high levels of OTA were mainly associated with the presence of wheat, barley, and wheat or corn gluten flour.

The presence of wheat, integral wheat, and wheat gluten flour is systematically associated with T2 levels above the threshold values between 8.4 and 30 µg/kg. The highest association values of these ingredients were found for T2 levels above a threshold value of 30 µg/kg (OR = 14.22, OR = 9.16, and OR = 9.20, respectively) (Figure S5a). Combinations of wheat with corn do not change the level of these associations (Figure S5b). Instead, the combinations of corn and beet or rice showed weaker associations only with low values of T2 toxin, suggesting that in the products we analyzed, corn content had a lesser contribution to the overall levels of T2 toxin compared to wheat (Figure S5b).

We also found a systematic association between the combination of integral corn and barley and T2 levels, and the highest values correspond to an association with T2 levels above the threshold value of approximately 30 µg/kg (OR = 32, RR = 4.831, *p* = 0.003) (Figure S5c). We also mention two other combinations of corn gluten flour with beer rice as well as corn gluten flour with wheat that were also systematically associated with T2 levels, the highest intensity of this association being related to T2 levels above 23 µg/kg and 30 µg/kg (OR = 38.5, RR = 5.682, *p* = 0.002 and OR = 32.0, RR = 12.987, *p* = 0.003, respectively) (Figure S5d).

## 4. Discussion

The most recent reviews and meta-analysis studies estimate the prevalence and concentration of mycotoxins in cereals and their derived products (bread, cornflakes, breakfast cereals, biscuits, crackers, and pasta-based products), and highlight the co-occurrence of mycotoxins in foods and feeds, their toxicity in various combinations (additive, synergistic and antagonistic effects), and the fungal source and geographical occurrence of mycotoxins [11,20,33,45,46,47]. Instead, our study focused on highlighting the associations between the ingredients (defined as variables in Table 3 and Table 4) of the investigated products and the mycotoxins levels. In addition, nowadays, it is a trend to use more than three types of ingredients to increase the nutritional value, fiber, mineral, and vitamin intake [48,49].

Most of the surveys from all over the world that have reported the co-occurrence of mycotoxins referred to the main mycotoxins AFs, OTA, ZEA, FUM, and DON that frequently contaminate cereals or derived foods [16,17,50]. However, the coexistence of mycotoxins must also be analyzed through the correlations between the levels of different types of mycotoxins found in cereals and dry food using the correlation coefficients to provide further consistency and significance to the study’s results. Applying such statistical analysis models, toxicity studies carried out in vitro and in vivo that used combinations of these mycotoxins would have a more realistic starting point regarding the level of mycotoxins and their combinations. The experimental doses were usually higher than those found in contaminated foods and often exceeded international regulatory limits [20].

The results of a meta-analysis study showed that out of 116 mycotoxin combinations found in cereal and derived cereal product samples, AFs + FUM, DON + ZEA, AFs + OTA, and FUM + ZEA were the most frequent, and DON, FUM, and ZEA are the most widespread mycotoxins in the world, with a prevalence of 66%, 56%, and 53% respectively [11]. Our results demonstrated that among the 34 products analyzed, only two presented a co-occurrence of four mycotoxins, the rest having detectable levels for all six mycotoxins studied (Table 5), having, with some exceptions, levels below the recommended limits [32], probably due to the extrusion process used [3,4]. Interestingly, the average level of AFs detected in the analyzed products (Table 5) was 1.6 times higher than those calculated in a meta-analysis study in the case of pasta, which had the highest values [45,47]. In the same meta-analysis, the highest average values of DON were recorded in the case of breakfast cereals, at 113 µg/kg and the average in the case of our study was 429 µg/kg (Table 5). Our study revealed that the median-level differences generated by the presence of wheat or corn gluten flour recorded the highest values for DON (272.13 and 129.22 µg/kg, Figure 2). In the meta-analysis mentioned above, bread had the highest average value of OTA (2.69 µg/kg), a mean level comparable to that recorded in our products (Table 5), as well as the difference in median levels of products containing integral wheat and those that do not contain this ingredient (Figure 2). Regarding ZEA, our study detected an average value of 90.9 µg/kg (Table 5), and the ingredients that contributed to this value were likely corn-based (Figure 2). This value was 2.45 times higher than the average value recorded in the case of cornflakes [45,47].

The correlation presented in Table 6 was confirmed by the analysis of the median-level differences generated by the presence of one or more ingredients (Figure 2). Our analysis showed that among the products that had the combination of corn and barley, there were significant statistical differences in the medians in the case of DON, FUM, T2, and OTA, with the latter probably mainly originating from barley and the other mycotoxins from corn (Figure 2). This is because barley has an exceptionally high likelihood of OTA contamination [11], and among cereal grains, DON, FUM, and ZEA mainly appear in corn [30]. In the case of toxin T2, the difference in the median may be explained by an additive effect when both ingredients (corn and barley) are combined in dry dog food (Figure 2b). This combination may also have a positive effect, reducing the level of ZEA in the case of products containing barley (Figure 2a). In the case of toxin T2, the additive effects were also highlighted in dry dog food containing corn gluten flour in combination with wheat, rice, or barley (Figure 2b).

Mycotoxins can be concentrated in different fractions of grains such as bran, flour, and shorts due to the milling process, which leads to an increased level of a mycotoxin in one fraction and a decreased level of that mycotoxin in another fraction [47]. Thus, it can be explained the significant increase in median value of OTA occurring in the case of the products containing corn gluten flour compared to those containing integral corn (Figure 2b), in addition to the median values of DON, FUM, and ZEA (Figure 2a). In this regard, several Fusarium strains producing ZEA also produced trichothecenes such as DON, and in general, a frequent co-occurrence of ZEA with other Fusarium toxins has been described in cereals, especially in corn [18,19,20,51]. Moreover, we found that the presence of wheat gluten flour was associated with the highest median differences for DON and AFs (Figure 2), which were also linked by a statistically significant correlation coefficient (Table 6). In addition, no significant correlation was found between AFs and OTA or toxin T2 (Table 6). Although except for one product, all products were simultaneously contaminated with AFs and OTA (Table 5), it can be observed that the two mycotoxins do not present statistically significant correlation coefficients (Table 6). Therefore, our findings highlight that the co-occurrence of mycotoxins should be discussed in terms of correlation coefficients and their statistical significance.

The associations shown in Figures S1–S5 confirm and complement the results presented in Figure 2, enriching them with important statistical significance. The higher levels of association do not mean changes in the effect of the added ingredients on the median-level differences already attributed to ingredients of the first or second category described in Section 2.4.3. The results in Figure 2 correspond, in Figures S1–S5, to the associations described by OR values between the presence of one or two ingredients and levels above the 50th percentile, $t_{\ell }^{50\%}$. However, Figures S1–S5 offer an extended picture showing all statistically significant associations (*p* < 0.05) between the presence of the ingredients and combinations of them shown in Figure 2 with levels of mycotoxins at the 50th percentile as well as other associations than those described by the threshold values, $t_{\ell }^{50\%}$. The systematic increase in the degree of association (OR and RR) found for as many threshold values, $t_{\ell }^{\alpha }$ (α>50%) is indicative of the additive role of the second ingredient in increasing mycotoxin levels. Thus, Figure 2 represents a snapshot of the associations between the presence of an ingredient or combinations of ingredients with values of a particular mycotoxin, ℓ, above a threshold value $t_{\ell }^{\alpha }$.

The study’s main limitation was related to the lack of transparency regarding the composition of the analyzed products. Even if the sources of the ingredients appear on the labels, their quantitative proportions are not specified (absolute value, upper limit value, or value range). In addition, derived ingredients (flour and cereal components) are not expressed consistently from one producer to another. The range of products we analyzed was also limited since in Romania, they are currently only available from three manufacturers. Finally, the exact storage and extrusion conditions that impact mycotoxin levels were not available.

## 5. Conclusions

As an ingredient source, corn was associated with high levels of DON and ZEA. In addition, the presence of corn gluten flour in the dry dog food composition was also associated with high levels of DON, FUM, ZEA, and OTA. Wheat was associated with high levels of DON and OTA, while the presence of wheat gluten flour was associated with high levels of DON and AFs. The methodology described in this paper suggests that a risk assessment should be conducted during the development of dry food recipes to identify the ingredient combinations that should be avoided due to their additive effects on mycotoxin levels and the ingredient combinations that reduce these levels. This analysis offers the opportunity to obtain even more rigorous results if the composition of the analyzed food products were specified in more detail, especially by the percentage of each ingredient. Hence, we forward the recommendation in general terms for greater transparency in the composition description of foods and feeds to ensure safety and quality criteria.

## Figures and Tables

**Table 1:** The number of products sampled from the ranges proposed by the three manufacturers for the three categories of diets.

	Diet Category			Total
	MC	AC	GC	
Producer A	3	4	7	14
Producer B	3	3	2	8
Producer C	3	3	6	12
Total	9	10	15	34

MC, metabolic conditions; AC, allergy conditions; GC, gastrointestinal conditions.

**Table 2:** The main ingredients of the studied dry dog food samples grouped by diet.

Diet Category	Corn *	Wheat *	Rice	Barley	Oat	Green Peas	Beet	Potatoes	Soy	Flax	Sorghum	Fibers	Oil
MC	9	8	2	4	2	3	8	2	3	3	0	3	6
AC	4	1	6	1	1	2	7	3	3	0	1	2	10
GC	14	8	15	5	2	2	12	0	2	2	1	0	11
Total	27	17	23	10	5	7	27	5	8	5	2	5	27

* Unspecified origin; MC, metabolic conditions; AC, allergy conditions; GC, gastrointestinal conditions.

**Table 3:** Secondary class ingredients retrieved from product composition labels.

Primary List Ingredients	Corresponding Secondary List Ingredients
Corn	integral corn, corn gluten flour *, and corn starch *
Wheat	integral wheat, wheat gluten flour *
Rice	rice seeds, brown rice, beer rice, and rice husks *
Oil	soybean oil, coconut oil, and vegetable oil

* The secondary ingredients are derived from a primary ingredient (source).

**Table 4:** Primary and secondary ingredient and mycotoxin levels-related variables.

Class	Notation	Description	Values
Primary, ingredient-related	$x_{i}^{\left(1\right)}$	Qualitative variables describing the presence of the ingredient i in the primary list (*i* is indexing the ingredients in the column heads of Table 2)	0—if the ingredient is absent 1—if the ingredient is present
	$x_{j}^{\left(2\right)}$	Qualitative variables describing the presence of the ingredient j in the secondary list (*j* is indexing the ingredients listed in Table 3)	
Secondary, ingredient-related	$x_{k}^{\left(3\right)}$	Qualitative variables describing the presence of a combination between two primary ingredients (e.g., corn and wheat), between one ingredient in the primary list and another from the secondary list (e.g., corn and wheat gluten flour), or between two ingredients in the secondary list (e.g., corn gluten flour, and integral corn); k is indexing all the possible combinations	0—if the combination is absent 1—if the combination is present
Primary, mycotoxin-related levels	$t_{\ell }$	Quantitative variables describing the mycotoxin levels (in µg/kg), ℓ is indexing the mycotoxins listed in the column heads of Table 5	$t_{\ell }\geq 0$
Secondary, mycotoxin-related levels	$t_{\ell }^{\alpha }$	Quantitative variable describing the mycotoxin levels for α percentile ($t_{\ell }^{\alpha }$) of the variable $t_{\ell }$; α takes the values $\left\{25\%,33\%,50\%,67\%,75\%\right\}$	0—if $t_{\ell }\leq t_{\ell }^{\alpha }$ 1—if $t_{\ell }>t_{\ell }^{\alpha }$

**Table 5:** Descriptive statistics and recommended limits of mycotoxins identified in the analyzed samples.

Statistics		DON [µg/kg]	FUM [µg/kg]	ZEA [µg/kg]	OTA [µg/kg]	T2 [µg/kg]	AFs [µg/kg]
Indexing (ℓ)		1	2	3	4	5	6
Mean		429.67	1221.02	90.89	2.66	17.46	8.82
Std. Deviation		171.08	1485.71	58.69	1.86	13.05	6.69
Variance		29,267.39	2,207,337.69	3444.94	3.48	170.33	44.70
Minimum		135.11	24.00	0.00	0.00	0.00	0.86
Maximum		839.21	7536.34	230.24	7.10	48.18	23.55
Coefficient of variation (%)		39.81	121.67	64.57	69.92	74.74	75.85
Percentiles	25	331.88	215.50	47.55	1.33	6.24	2.72
	33	364.04	609.21	55.39	1.44	8.41	3.37
	50	403.06	962.42	82.53	1.76	13.75	8.02
	67	462.60	1097.56	112.48	3.12	23.10	11.52
	75	502.40	1189.70	136.88	3.93	29.87	13.85
Recommended limit (in µg/kg) [32]		5000	5000	100/200	10	50	10
Beyond the recommended limit (N)		0	1	14	0	0	12
%		0	2.9	41.2	0	0	35.3

DON, deoxynivalenol; FUM, fumonisins; ZEA, zearalenone; OTA, ochratoxin; T2, T-2 toxin; AFs, aflatoxins.

**Table 6:** Correlation matrix of mycotoxin levels containing Spearman coefficients and two-tailed statistical significance.

	AFs [µg/kg]	DON [µg/kg]	FUM [µg/kg]	OTA [µg/kg]	T2 [µg/kg]	ZEA [µg/kg]
AFs [µg/kg]	1	0.488 ** 0.003	0.368 * 0.032	0.176 0.32	0.185 0.296	0.352 * 0.041
	DON [µg/kg]	1	0.635 ** <0.001	0.343 * 0.047	0.481 ** 0.004	0.510 ** 0.002
		FUM [µg/kg]	1	0.437 ** 0.01	0.152 0.389	0.507 ** 0.002
			OTA [µg/kg]	1	0.436 ** 0.01	0.329 0.057
				T2 [µg/kg]	1	0.368 * 0.032
					ZEA [µg/kg]	1

** Correlation is significant at the 0.01 level (2-tailed), in light pink; * Correlation is significant at the 0.05 level (2-tailed), in light green. DON, deoxynivalenol; FUM, fumonisins; ZEA, zearalenone; OTA, ochratoxin; T2, T-2 toxin; AFs, aflatoxins.

## Data Availability

Not applicable.

## Supplementary Materials

The following supporting information can be downloaded at: https://www.mdpi.com/article/10.3390/foods12010110/s1, Figure S1: Association levels of ingredients or combinations of ingredients with DON levels above the threshold values, $t_{1}^{\alpha }$; Figure S2: Association levels of ingredients or combinations of ingredients with FUM levels above the threshold values, $t_{2}^{\alpha }$; Figure S3: Association levels of ingredients or combinations of ingredients with ZEA levels above the threshold values, $t_{3}^{\alpha }$; Figure S4: Association levels of ingredients or combinations of ingredients with OTA levels above the threshold values, $t_{4}^{\alpha }$; Figure S5: Association levels of ingredients or combinations of ingredients with toxin T2 levels above the threshold values, $t_{5}^{\alpha }$.
