# Supplementary material for: Composition-Based Risk Estimation of Mycotoxins in Dry Dog Foods
Source: Foods. 2022 Dec 25;12(1):110. doi: 10.3390/foods12010110 (PMC9818488; doi:10.3390/foods12010110)
Supplement: Supplementary file 1 [file foods-12-00110-s001.zip › foods-2091310-supplementary.pdf]

Supplementary materials

Composition-Based Risk Estimation of Mycotoxins in Dry Dog Foods

Ovidiu Ionut Geicu [1,†], Liviu Bilteanu [1,2,†], Loredana Stanca [1], Adriana Ionescu Petcu [1], Florin Iordache [1], Aurelia Magdalena Pisoschi [1] and Andreea Iren Serban [1,3,*]

1 Department of Preclinical Sciences, Faculty of Veterinary Medicine, University of Agronomic Sciences and Veterinary Medicine of Bucharest, 105 Blvd. Splaiul Independentei, 050097 Bucharest, Romania
2 Molecular Nanotechnology Laboratory, National Institute for Research and Development in Microtechnologies, 126A, Erou Iancu Nicolae Street, 077190 Bucharest, Romania
3 Department of Biochemistry and Molecular Biology, Faculty of Biology, University of Bucharest, 91–95 Blvd. Splaiul Independentei, 050095, Bucharest, Romania
* Correspondence: irensro@yahoo.com; Tel.: +40-021-3180469; Fax: +40-021-3180498
† These authors contributed equally to this work.

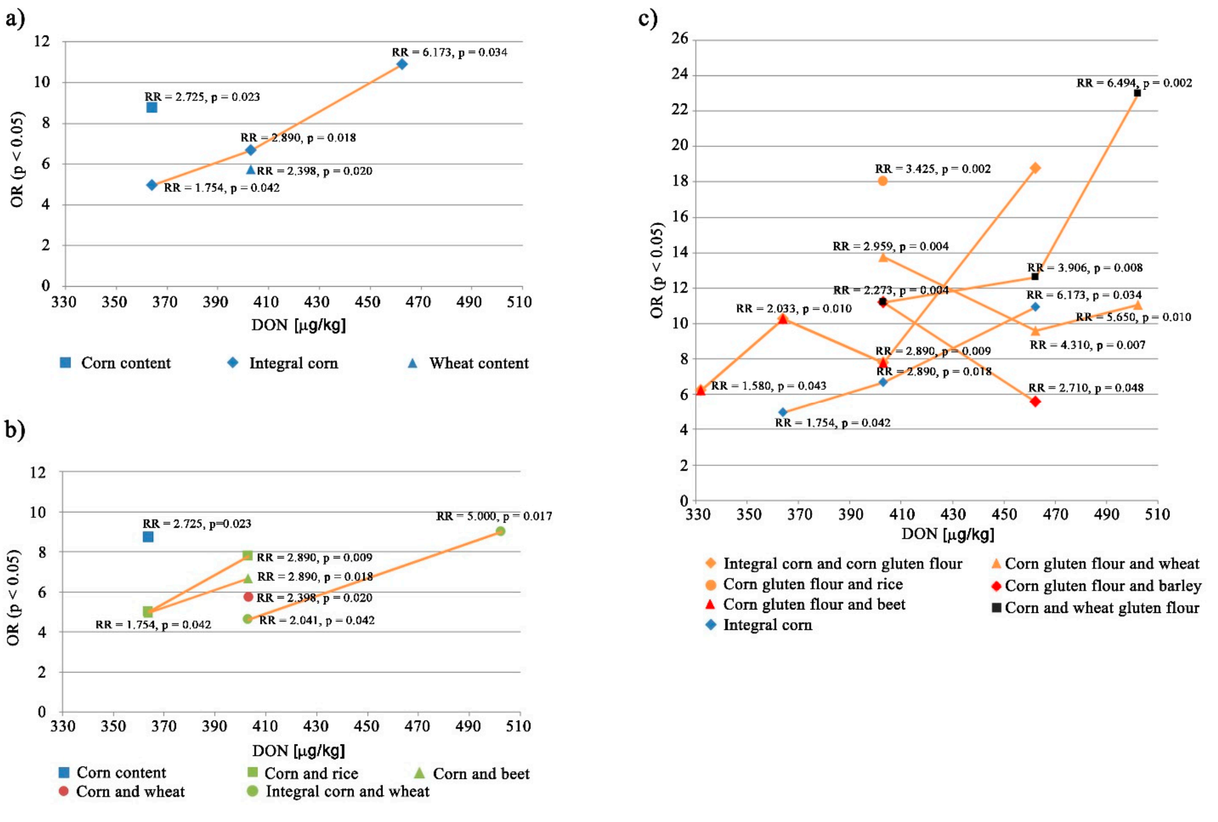

Figure S1. Association levels of ingredients or combinations of ingredients with DON levels above the threshold values, t_1^{α}: (a) corn, integral corn, and wheat; (b) combinations of corn with other ingredients; (c) combinations of corn or wheat gluten flour with other ingredients. We kept the integral corn for reference in (b) and (c).

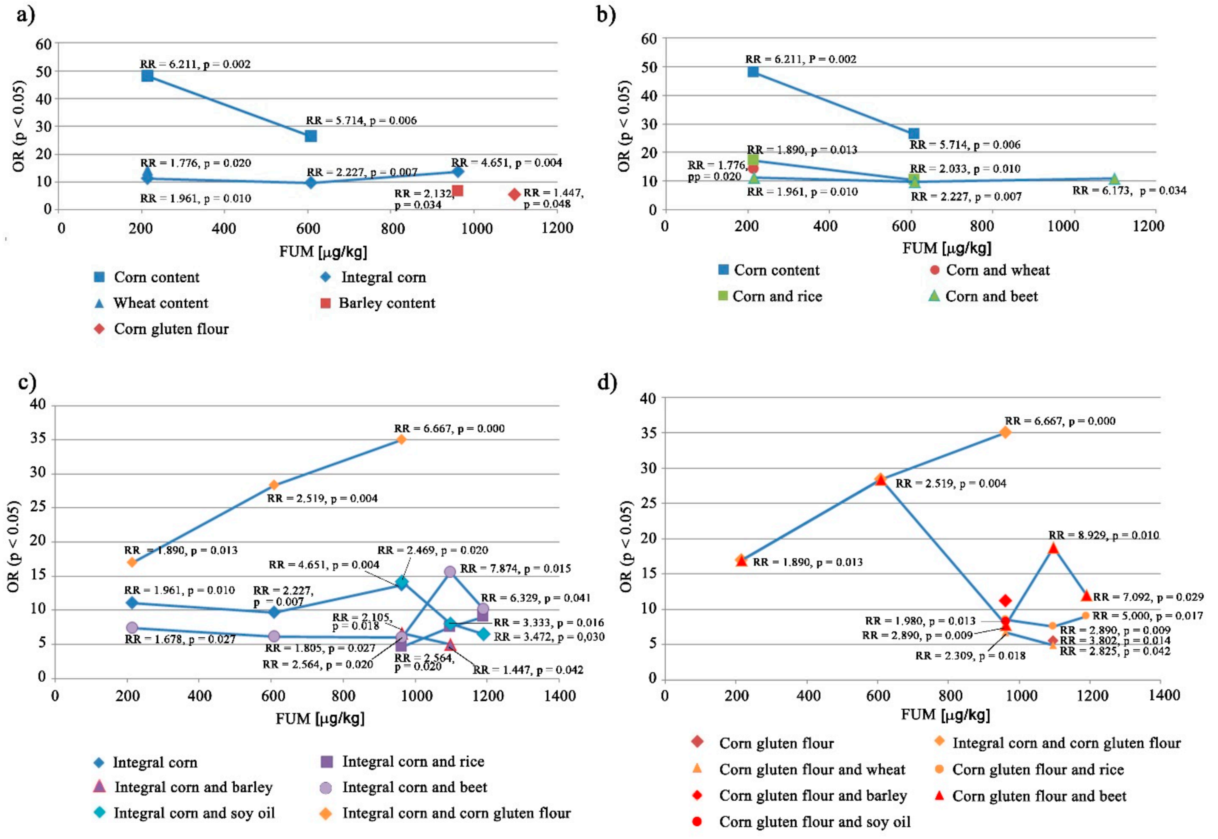

Figure S2. Association levels of ingredients or combinations of ingredients with FUM levels above the threshold values, t_2^{α}: (a) corn, integral corn, wheat, barley, and corn gluten flour; (b) combinations of corn with other ingredients; (c) combinations of integral corn with other ingredients; (d) combinations of corn gluten flour with other ingredients. We added the corn, the integral corn, and corn gluten flour for reference in (b), (c), and (d), respectively.

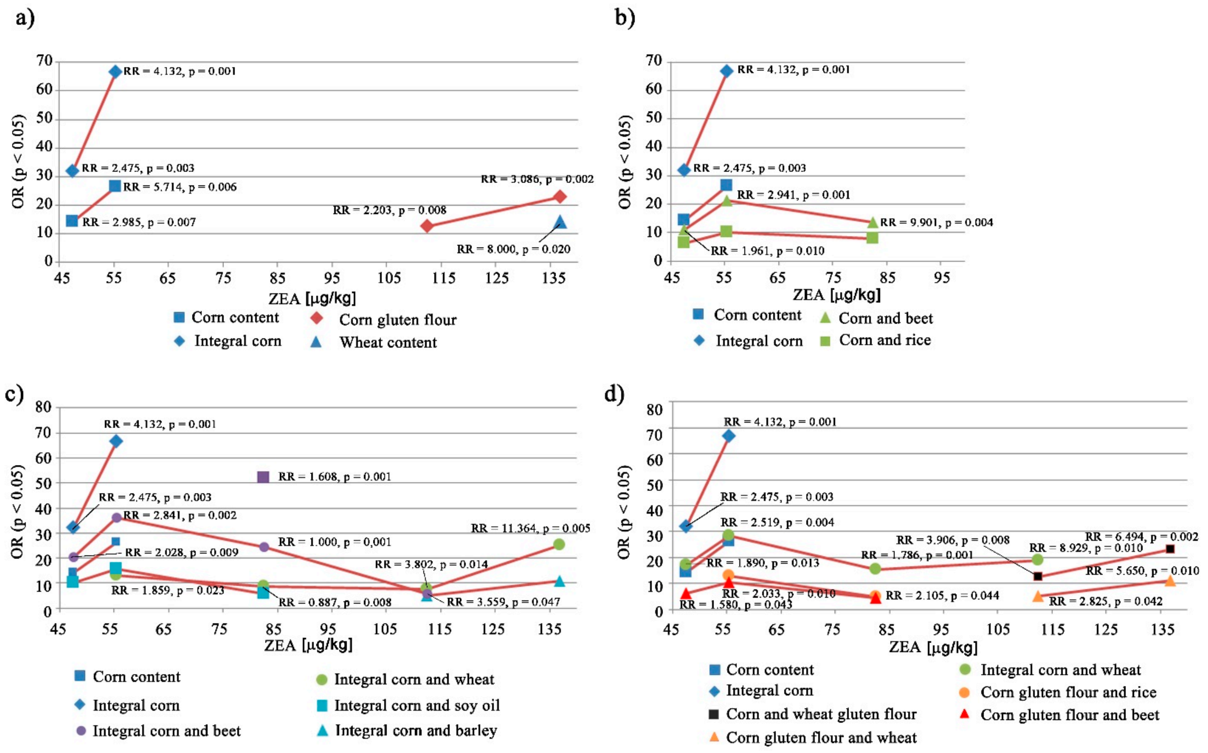

Figure S3. Association levels of ingredients or combinations of ingredients with ZEA levels above the threshold values, t_3^{α}: (a) corn, integral corn, wheat, and corn gluten flour; (b) combinations of corn with other ingredients; (c) combinations of integral corn with other ingredients; (d) combinations of corn or wheat gluten flour with other ingredients. We added the corn and the integral corn for reference in (b), (c), and (d).

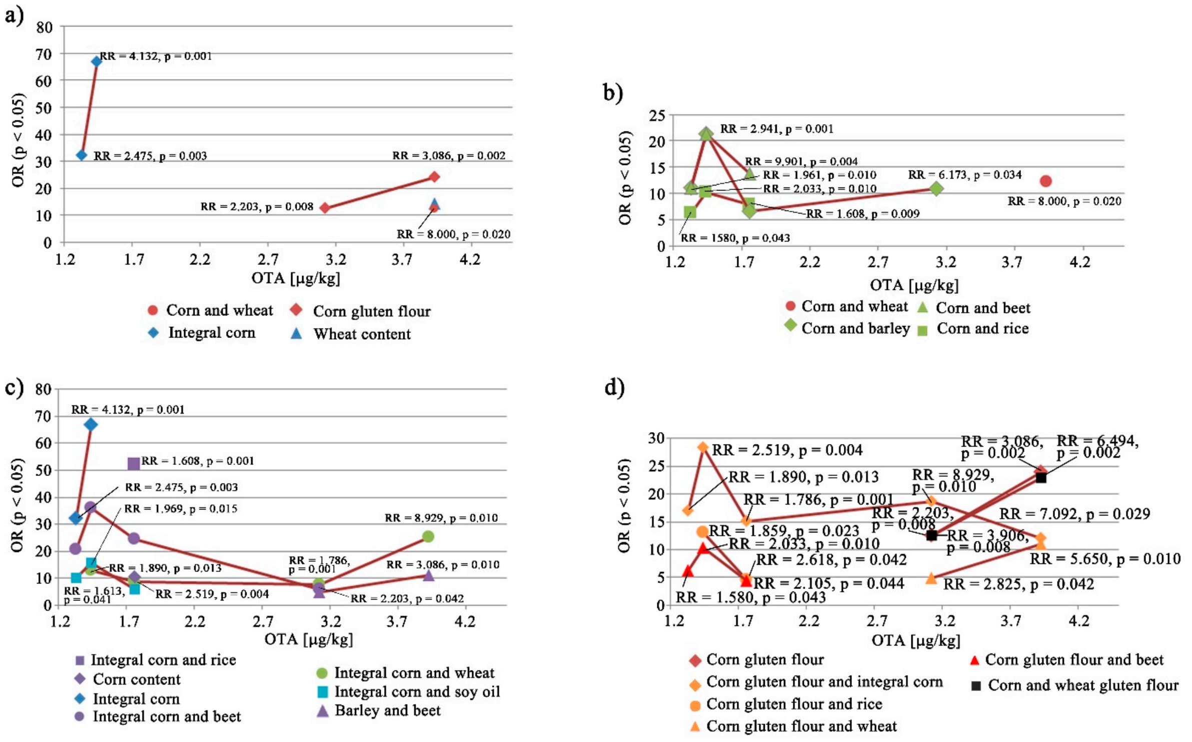

Figure S4. Association levels of ingredients or combinations of ingredients with OTA levels above the threshold values, t_4^{α}: (a) integral corn, wheat, corn and wheat and corn gluten flour; (b) combinations of corn with other ingredients; (c) combinations of integral corn with other ingredients; (d) combinations of corn or wheat gluten flour with other ingredients. We added the corn and the integral corn for reference in (c).

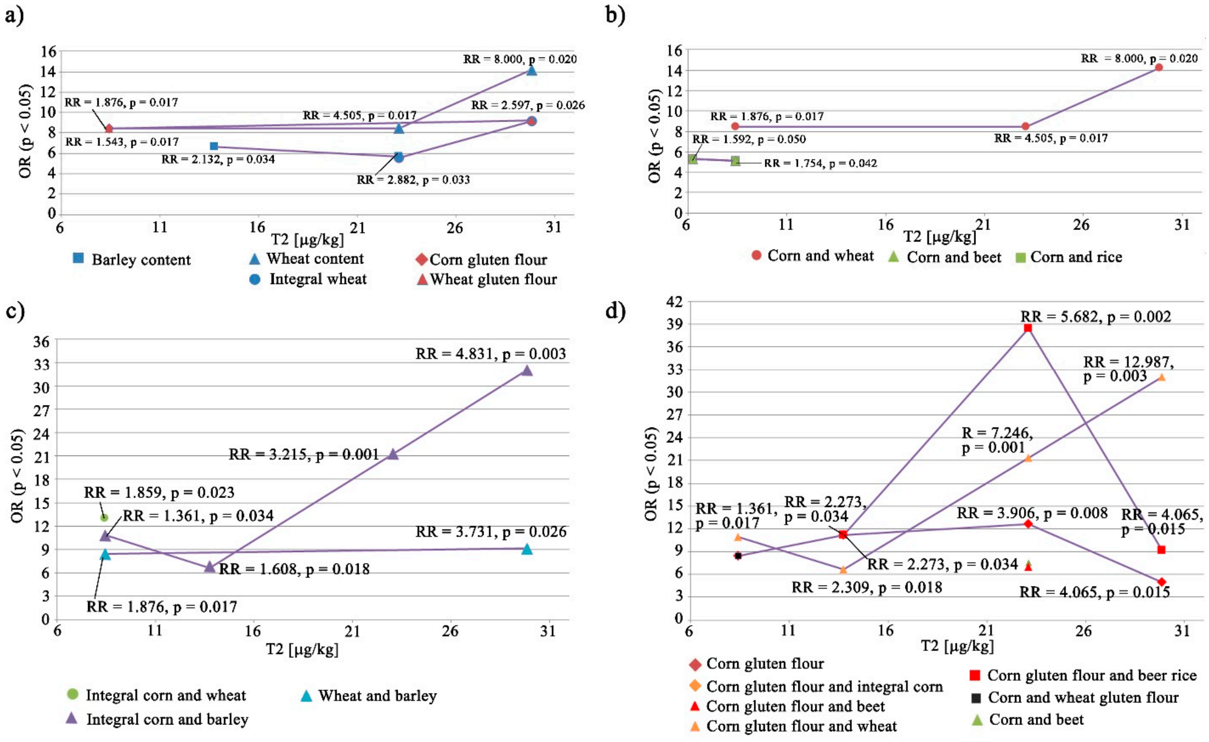

Figure S5. Association levels of ingredients or combinations of ingredients with T2 levels above the threshold values, t_5^{α}: (a) integral corn, wheat, integral wheat, barley, corn and wheat gluten flour; (b) combinations of corn with other ingredients; (c) combinations of integral corn with other ingredients; (d) combinations of corn or wheat gluten flour with other ingredients.
